# Analysis of surveillance and prevention plan for African Swine Fever in Italy in 2020

**DOI:** 10.1002/vms3.824

**Published:** 2022-06-08

**Authors:** Carmen Iscaro, Valentina Cambiotti, Olivia Bessi, Francesca Pacelli, Luigi Ruocco, Francesco Feliziani

**Affiliations:** ^1^ Istituto Zooprofilattico Sperimentale Umbria e Marche: Laboratorio Nazionale di Referenza per le Pesti Suine Perugia Italy; ^2^ Ministero della Salute: Direzione Generale della Sanità Animale e dei Farmaci Veterinari, ufficio III, Sanità animale e gestione operativa del Centro nazionale di lotta ed emergenza contro le malattie animali e unità centrale di crisi Roma Italy; ^3^ Istituto Zooprofilattico Sperimentale Umbria e Marche: Sistema Informatico, Informatizzazione dei Servizi Sanitari Perugia Italy; ^4^ Ministero della Salute: Direzione Generale per l'Igiene e la Sicurezza degli Alimenti e la Nutrizione, ufficio VIII, Sistema di allerta, emergenze sanitarie e pianificazione dei controlli Roma Italy

**Keywords:** African Swine Fever, domestic pigs, early detection, surveillance, wild boars

## Abstract

**Background:**

African Swine Fever (ASF) is a challenge for pig health worldwide. The disease has spread to multiple countries on five continents. ASF‐free countries need to apply effective strategies to prevent the introduction of infection.

**Methods:**

Italy implemented a surveillance and prevention plan for ASF in 2020, supported by a dedicated information system. Several pillars for action have been identified: passive surveillance in both domestic pigs and wild boar populations, verification of the implementation of biosecurity measures on farms and an awareness campaign for all involved stakeholders.

**Results:**

There were some regional differences in the management of passive surveillance. In order to identify all critical points and apply corrective measures, regional authorities were called to carry out a gap analysis exercise in July 2020. There were an adequate number of samples collected from wild boar but the number of samples collected from domestic pigs was below the target in most regions. Furthermore, sample distribution within the country was not homogeneous.

**Conclusions:**

During the forthcoming year, some issues must be addressed in order to establish an effective early detection system in Italian ASF‐free regions.

## Graphical abstract

African Swine Fever (ASF) is a challenge for pig health worldwide. Italy implemented a surveillance and prevention plan for ASF in 2020. An analysis of results of this plan for 2020 has been carried out.

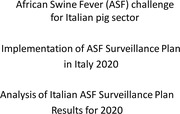

## 1 INTRODUCTION

African Swine Fever (ASF) is a haemorrhagic disease that occurs in domestic pigs and wild boars. It is caused by a DNA virus belonging to the *Asfarviridae* family. ASF was first detected in sub‐Saharan Africa, where it is an endemic disease maintained by a wild cycle between warthogs and *Ornithodoros* soft tick vectors, but it was able to spread worldwide, both by direct as well indirect contact (Blome et al., 2020). Clinical symptoms in host species include high fever, anorexia, respiratory and gastrointestinal signs, abortion, ataxia, haemorrhagic symptoms, and in experimental infections with highly virulent strains, the mortality rate can reach 100% (Blome et al., 2012; Guinat et al., 2016). Although it is not a zoonotic disease, its importance is due to its high case fatality rate and ongoing spread (Schulz et al., 2019), which can cause severe economic losses in affected countries.

Moreover, the virus is very stable in the environment and in food, being able to remain in preserved meat for up to 6 months (Sánchez‐Vizcaíno et al., 2009), leading to the possibility of the virus being transmitted, especially to wild boars, through abandoned human food waste (Farez & Morley, 1997; McKercher et al., 1978; Mebus et al., 1993; Mebus et al., 1997). For all these reasons, it is classified as a notifiable disease and listed in the Terrestrial Animal Health Code (OIE, 2019) and European Animal Health Law (AHL) (Regulation EU, 2016). ASF, originating on the African continent, reached Georgia in 2007, spread to the Asian continent, arrived in the European Union in 2014, in China in 2018, in Papua New Guinea and India in 2020 (Blome et al., 2020). Furthermore, the first ASF outbreak in the Americas in the last 40 years was reported in July and September 2021 in the Dominican Republic and Haiti (USDA, 2021a; USDA, 2021b). In the first half of 2021, ASF has been reported by the following European countries: Bulgaria, Estonia, Germany, Hungary, Italy (in the Sardinia Region only), Latvia, Lithuania, Moldova, Poland, Romania, Serbia, Slovakia and Ukraine (ADIS, 2021). Among these countries, Italy has experienced a peculiar situation because ASF has been present in the Sardinia Region since 1978, probably due to the introduction of contaminated food waste from the Iberian Peninsula (Laddomada et al., 2019), while the rest of the country is free of the disease.

In the Italian Peninsula, the risk of ASF introduction arises primarily from human factors related to contact with infected areas in different countries. The risk of importation from the Sardinian infected area is quite lower than the risk of importation from other countries because Sardinia is isolated from mainland Italy and rigorous controls at ports and airports, as well as application of regionalisation guidelines to pig products, protect ASF‐free zones from ASF virus introduction. Moreover, in recent years the risk of infection on the island has decreased considerably, with evidence of viral circulation reduction during the past 2 years, after specific measures were implemented to reduce the prevalence of infection in the endemic area. So the risk of ASF introduction from Sardinia is lower than in the past (Cappai et al., 2020).

Nevertheless, the introduction of contaminated food or materials transported by travellers is difficult to prevent, avoid, and, more importantly, is not predictable. For these reasons, in 2019, the Italian Ministry of Health, supported by the National Reference Laboratory for Swine Fevers (NRL), developed a surveillance plan to be implemented in 2020, with the aim of preventing the possible introduction of virus in the rest of the country through early detection of infection (Italian Ministry of Health, 2019). This plan was presented for the approval of the European Union Commission as a part of a more extensive document that included the annual ASF eradication plan for the Sardinia Region.

Therefore, the aim of this paper is to describe the implementation of the Italian surveillance and prevention plan in ASF‐free territories, present the initial results and describe the gaps identified after the first year of its implementation.

## 2 MATERIALS AND METHODS

In 2020, the Italian Ministry of Health implemented a national surveillance and prevention plan to prevent and detect ASF infection in domestic pigs and wild boars. The plan included different actions, such as passive surveillance measures in wild boar populations and domestic pigs, application of biosecurity measures, and communication and training of stakeholders. The surveillance program was based on collecting and analysing samples from found dead animals and on the notification of every suspect case. National legislation, as recommended by European rules, requires that any suspect case of ASF should be notified; moreover, if a suspect case of ASF is detected, the National Contingency Plan should be applied. The measures required to prevent the spread of infection are maintained by the local veterinary service until laboratory investigations enable the occurrence of the disease (both ASF and classical swine fever) to be ruled out. In the event that an outbreak is confirmed, all the measures provided by the National Contingency Plan are confirmed and reinforced in the restricted zones (surveillance and protection zones) around the first detection of the virus (http://www.salute.gov.it/imgs/C_17_pagineAree_1670_listaFile_itemName_0_file.pdf).

Regarding passive surveillance in wild boars, everyone is required to report discoveries of carcasses found in the field, and the local veterinary service collects samples from these animals (including wild boars killed in motor vehicle accidents). Wild boar sampling was planned in order to rule out ASF virus infection in the general wild boar population. The ASF plan includes the sampling of all wild boars found dead; in order to provide a number to be used as indicator in reporting, a minimum target of 600 samples is identified for the whole country. Each Italian region is required to collect a certain number of samples, according to the wild boar population estimate in that territory, with the aim to find at least one positive sample in case of infection presence. Each regional target represents in turn the minimum number of samples to be collected and tested. Anyway, of course, in case of reasonable suspicion of ASF based on the clinical signs, post mortem lesions, or epidemiological evidence (such as several dead wild boars found in the same area), the National Contingency Plan should be applied.

Regarding domestic pigs, any suspected case (such as mortality rate over 30%, presence of haemorrhagic clinical signs or lesions) must be notified to the competent authority, and the authority should apply the measures described in the specific contingency plan. Sampling for passive surveillance in domestic pigs was planned for all suspect cases and preferably for farms including less than 50 animals (family and backyard farms). In the absence of suspect cases, veterinary service is required to collect samples from at least two dead pigs per region and per week, in order to improve the sampling sensitivity.

Moreover, in order to include the rearing systems considered at major risk for the transmission of ASF due to their lower levels of biosecurity, in every region, half of the whole amount of samples planned from domestic pigs should be collected from backyard and family farms, while the remaining ones should be collected from commercial farms.

For both wild boars and domestic pigs, the collected target organs, such as the spleen, kidney, lymph nodes, or bone in the case of wild boar carcasses in an advanced state of decomposition, are delivered to the competent official laboratory for analysis. The veterinary service can choose to send organs or the whole carcass to the laboratory, according to the regional guidelines. Samples collected for surveillance are accompanied by a dedicated form reporting information about the animal (age, sex), the location where it was found (geographical coordinates for wild boars) or origin farm details (for domestic pigs) and the sample type (organ).

All forms are registered in a national database, hosted by the national portal of the animal information system (Vetinfo), and is used for both wild boars and domestic pigs. The database contains all data regarding each sample collected for ASF passive surveillance. Specifically, the veterinary services are responsible for data entry regarding farm details (domestic pigs) and source location (wild boars), while laboratories input the results of the diagnostic tests. In fact, each form is identified by a unique numeric code that allows the sample to be traced and matches the sample identification data with the corresponding results.

The collected samples are used for ASF genome investigation in line with the European Commission guidelines on rapid alert systems (Working Document SANTE, 2015). Routine tests are carried out by the network of the Istituti Zooprofilattici Sperimentali regional laboratories, while confirmatory tests are examined by the NRL. In the event of any clinically or epidemiologically suspected case, samples are sent directly to the NRL. All laboratories were asked to take part in a ring test, arranged by the NRL, so that standard diagnostic procedures could be followed throughout the country. The NRL suggested the use of real‐time PCR targeting a 250 bp fragment of the reference strain BA71V. It represents an extremely conserved portion of the B646L gene, which encodes the principal structural protein of the virus (VP72) and is able to identify all known genotypes (King et al., 2003). A few laboratories chose to apply other protocols using commercial kits that complied with international standards.

NRL supports the Ministry of Health compiling periodic reports to verify the progress of surveillance activities for ASF and to manage any critical issues. A general activity check was conducted from June to September 2020, by engaging with regional authorities. The gap analysis approach was used to evaluate eventual gaps affecting surveillance activities and to identify corrective measures. Specifically, 20 regional working groups have been established, including regional veterinary authorities, that focused on the territory under their jurisdiction.

The whole process was planned in three stages. First, a virtual meeting was held, after the first semester of plan implementation, on June 2020. Ministry of Health described the aim of this verification activity and asked each regional delegation to conduct a gap analysis based on a standardised questionnaire. Then regional working groups carried out their assignments in three months. In order to perform the required activities, components of each group met as often as needed, and they could be supported by NRL in those occurrences more difficult to solve. When completed, gap analysis results were evaluated by Ministry of Health and NRL for each region. Finally, a virtual meeting took place on September 2020 in order to give a feedback to working groups. So, based on gap analysis results, adequate measures were recommended by Ministry of Health in order to solve the identified regional gaps.

The questionnaire comprised five key sections about the implementation of the ASF plan: passive surveillance in wild boars, passive surveillance in domestic pigs, wildlife management procedures, implementation of biosecurity measures, and training activities. For each topic, the regional working groups were asked to provide the following information:

‐ The current situation and optimal standards to be achieved
‐ Identification of gaps between the current and the desired situation and of the measures to be applied
‐ Evaluation and identification of the resources needed to fill the gaps identified
‐ Identification of factors that could promote or limit performance of surveillance activities

## 3 RESULTS

The law orders were not yet ready at the beginning of 2020 and the official launch of the plan was delayed. In addition, the national database was implemented later than the official launch of the plan. For this reason, samples collected in the first months of 2020 were registered in the database retrospectively, and some data may have been lost.

Therefore, many of the records were non‐compliant, registering incomplete data, both for domestic pigs and for wild boar. In particular, a considerable number of samples were not registered correctly, lacking some data in the database, for different reasons, such as:

- Veterinary services accidentally creating a record, resulting in the database having an incomplete form suggesting that samples have not been provided
- Regional laboratories failing to enter test results, resulting in the database having an incomplete form suggesting that tests have not been conducted

This topic was accentuated especially in some regions, where data entry work proved to be more difficult, for insufficient skill or matter of delay in data input.

**FIGURE 1:**
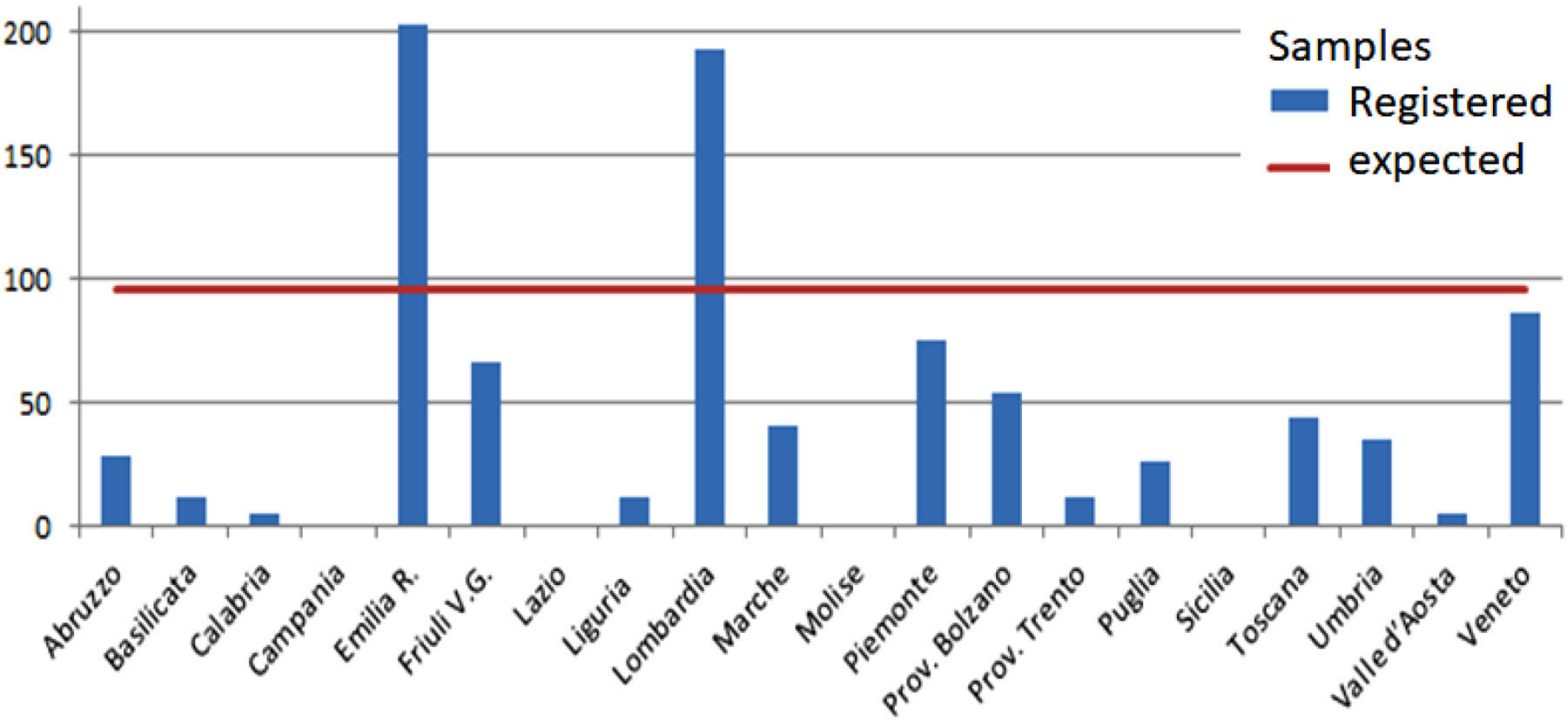
National surveillance for swine fever in domestic pigs in Italy. The bar chart shows the number of samples correctly registered in the National Database (blue columns) compared to the target number of samples (red line). The data were extracted from the National Information System in February 2021 and covered the period from 01‐01‐2020 to 31‐12‐2020

Regarding domestic pigs, the target was reached in only a few regions (Figure 1). Certain regions collected and registered a very low number of samples. Moreover, in other cases, pigs were properly sampled as planned, but the data were not registered in the national database.

**FIGURE 2:**
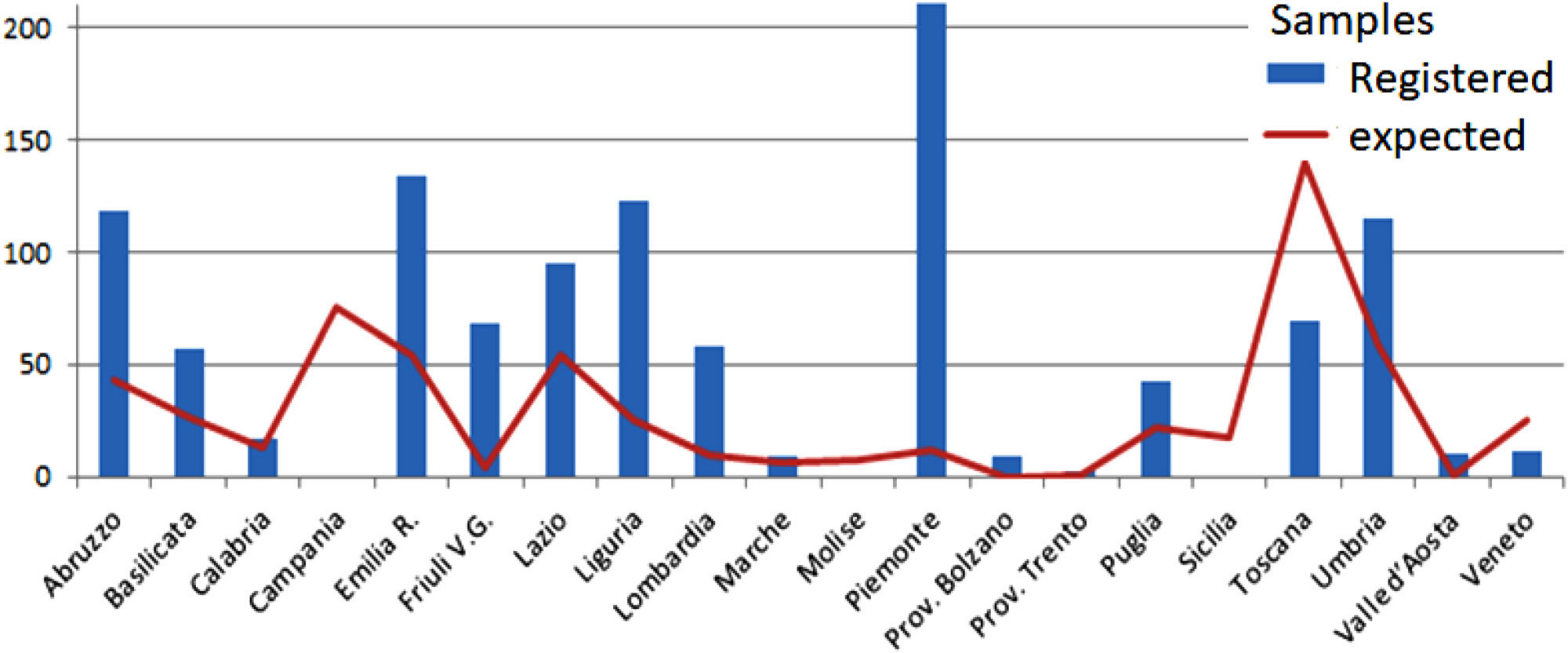
National surveillance for African swine fever in wild boars in Italy. The bar chart shows the number of samples correctly registered in the National Database (blue columns) compared to the target number of samples (red line). The data were extracted from the National Information System in February 2021 and covered the period from 01‐01‐2020 to 31‐12‐2020

Regarding wild boars, sampling started slowly in several regions, and many records were incorrectly or incompletely registered in the national database. Despite the difficulties, at the end of the year, many regions reached or exceeded the minimum number of samples requested by the plan. Specifically, 15 regions were able to achieve their sampling targets for wild boars (Figure 2), compared to the total number of regions in Italy (20). Although the information is not recorded in the national database, the majority of the wild boars examined were victims of vehicular collisions (personal communication from veterinary regional services).

**FIGURE 3:**
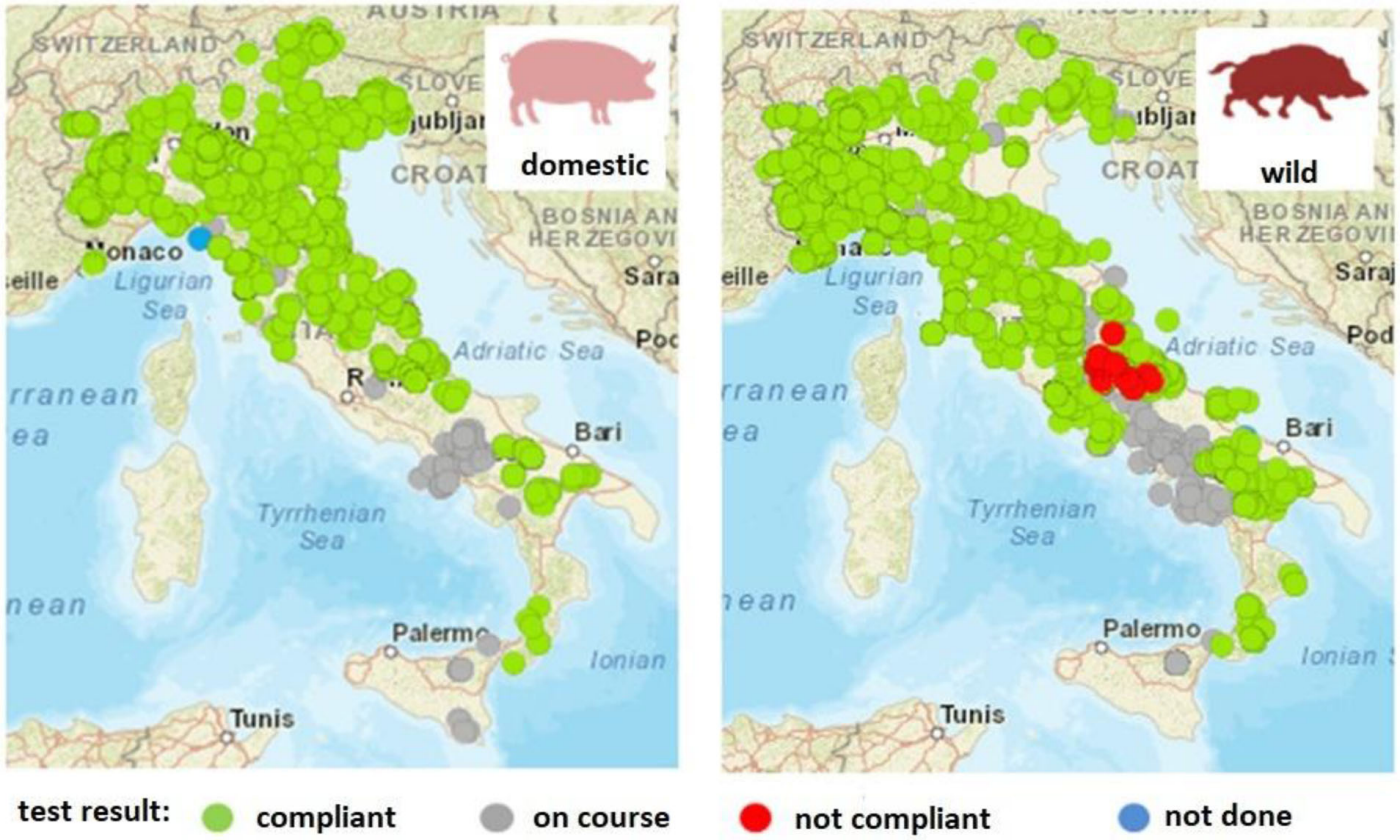
Number of registered samples collected from domestic pigs (left) and wild boars (right) according to geographic location for Italian passive surveillance of African swine fever (2020). The data were extracted from the National Information System in February 2021

Considering the localisation of sampled animals, wild boars were fairly homogeneously distributed throughout the country, but with fewer samples submitted in the southern regions. The under‐sampling of the southern regions was more marked for domestic pig surveillance (Figure 3). However, several issues were identified in the gap analysis exercise, such as a delay in the implementation of operative procedures, poor collaboration at the regional level among the different sectors involved, inadequate awareness of ASF in farmers and postponed or cancelled training events because of the COVID‐19 pandemic, which could explain the low compliance with the plan, at least in the first part of the year. Moreover, different regional working groups approached the gap analysis exercise differently: for example, certain regions identified practical measures to address the gaps whereas other regional working groups experienced difficulty not only in identifying the gaps but also in solution‐finding. Anyway, for each detected and discussed critical point, an appropriate action was recommended to fill the gap in that territory.

## 4 DISCUSSION

The Italian veterinary services have had the opportunity to get to know ASF very well. They have had longstanding experience because of the endemic situation in the Sardinia Region, which has required significant resources, particularly in recent years, to get closer to eradicating the infection and which still necessitates restrictions on the Sardinian swine sector effectively preventing its development (Loi et al., 2019).

Considering the evidence recorded over more than 40 years, the Italian and Sardinian authorities have demonstrated that they are able to effectively manage the risk of virus escape from Sardinia. There has been only one outbreak originating from Sardinia that occurred in 1983. It was notified and immediately eradicated (Danzetta et al., 2020).

At present, based on different conditions in Sardinian scenario rather than the European one (Laddomada et al., 2019; Loi et al., 2020; Mur et al., 2017), the threat of introduction of genotype II ASF virus from other countries in which ASF is endemic appears to be much more relevant, than the threat of outbreaks originating from Sardinia (Loi et al., 2019). Persistent infection in the wild boar population and the possibility of indirect transmission by human activities pose the greatest risk factors for the introduction of infection in ASF free regions (EFSA, 2014).

Thus, all Italian regions are exposed to the risk of ASF outbreaks that would have devastating effects on local and national economies. A possible ban on the export of fresh and cured pork products is a particular concern. Italian pork products are in large demand worldwide and are of great economic importance, with potential for growth in the near future.

For these reasons, the initiative of Ministry of Health to provide a dedicated surveillance plan, able to implement the actions required to prevent the spread of the disease in the wild boar population and the domestic swine population, was theoretically appreciated by stakeholders. The National Surveillance Plan applied in 2020 aims to promote greater awareness that the early detection of infection is necessary in order to reduce ASF serious health and economic consequences. In fact, the main objective of the plan is to manage the alert and pre‐emergence phases in order to increase the attention level in official veterinarians and farmers.

Overall, this plan is an ambitious programme in Italian contest. For the first time, the veterinary services had to consider wildlife as a relevant target of their activity. Similarly, the surveillance of domestic pigs has been planned in detail and is directed specifically towards small farms that often have an inadequate level of biosecurity. The plan was developed as the result of a collaboration between the Ministry of Health, the NRL, wildlife experts, and representatives of the regional authorities. In addition, in the past, disease eradication plans were based on active surveillance and detection of residual infected animals on farms, in order to obtain the indemnity status. In this case, however, the program is based on prevention and the protection of territories that are currently free of infection. This approach is perfectly in compliance with the guiding principles of the European AHL.

Despite these positive expectations and a spirit of active collaboration, the initial phases of the plan have revealed a number of implementation challenges, particularly in the first part of the year. General guidelines were not immediately received and implemented at local level and, in the absence of specific local procedures, an insufficient number of samples were collected, particularly in the southern regions.

Regarding the wild boar surveillance, most tested samples were related to road accidents. As these data were not officially recorded in 2020, it was established to insert the flag ‘wild boar road killed’ into the national database for 2021 and 2022 years. Nevertheless, the method of sampling wild boars needs further consideration because using samples collected following road accidents is a matter of debate (Jori et al., 2020).

Surveillance in domestic pigs was initially directed towards family and backyard farms, recognised as having the highest risk of exposure to the eventual introduction of the virus. However, family farms are intended for self‐consumption: mortality rate is low and farmers do not communicate data about mortality events regularly to veterinary services. For this reason, in the plan, also commercial herds were considered as feasible to be sampled for surveillance purposes.

Based on this evidence, the Ministry of Health, in cooperation with NRL, after the first half year of plan measures implementation, carried out an intensive verification activity by using the gap analysis technique, as above described. The impact of this training was positive and resulted in an increase in the number of samples submitted in the second half of the year.

However, some weaknesses remain unresolved. Specifically, sampling is not representative of the entire country because program administrators in some regions entered poor quality data in the surveillance system. This lack of data was partially due to a large number of incomplete records. Moreover, the COVID‐19 pandemic affected the organisation and outcome of the surveillance plan. Although ASF passive surveillance was considered a non‐deferrable activity, a number of regions requested a reduction of the targets specified in the plan.

Despite the difficulties, most of the Italian regions reacted in a positive manner to the surveillance plan during its first year of implementation, and as a result of this experience, compliance with the plan is likely to improve, and to reach an adequate level of surveillance in relation to the disease risk by the end of 2021. The ASF plan for 2021/2022 intends to increase the level of alerts and improve the early detection system. New tools, such as guidelines on the management of the wild boar population and a method to perform a risk analysis for passive surveillance in domestic pigs, have been introduced in the ASF plan for 2021/2022. It also includes also new activities, such as simulation exercises and carcass research.

The Italian Veterinary System hopes in this way to address the threat posed by ASF and to limit the damage of a possible future introduction of the infection in territories that are currently still ASF free.

Post scriptum: under the surveillance activities above described, on January 2022, ASF virus, genotype II, was detected in a wild boar found dead in Piedmont region. In mainland Italy, ASF threat has been real ever since.

## CONFLICT OF INTEREST

The authors declare no conflict of interest.

## ETHICS STATEMENT

The authors confirm that the ethical policies of the journal, as noted on the journal's author guidelines page, have been adhered to. No ethical approval was required as this article reports on routine surveillance activities.

## AUTHOR CONTRIBUTIONS

Writing‐ original draft preparation: V.C. Conceptualisation and writing: C.I. and F.F. Investigation and formal analysis: C.I. Review and editing: F.F. Visualisation: O.B., F.P., L.R. Supervision: F.F.

### PEER REVIEW

The peer review history for this article is available at https://publons.com/publon/10.1002/vms3.824

## Data Availability

N/A.

## References

[vms3824-bib-0001] ADIS (Animal Disease Information System) . (2021). Summary of the number of outbreaks and the date of the last outbreak notified to the European Union, Total Outbreaks per Disease, from 02.01.2021 until 29.09.2021. https://ec.europa.eu/food/system/files/2021‐10/ad_adns_outbreaks‐per‐disease.pdf (Accessed 19 October 2021)

[vms3824-bib-0002] Blome, S. , Franzke, K. , & Beer, M. (2020). African Swine Fever – A review of current knowledge. Virus Research, 287, 198099. 10.1016/j.virusres.2020.198099 32755631

[vms3824-bib-0003] Blome, S. , Gabriel, C. , Dietze, K. , Breithaupt, A. , & Beer, M. (2012). High virulence of African swine fever virus Caucasus isolate in European wild boars of all ages. Emerging infectious diseases, 18(4), 708. 10.3201/eid1804.111813 22469497 PMC3309674

[vms3824-bib-0004] Cappai, S. , Rolesu, S. , Feliziani, F. , Desini, P. , Guberti, V. , & Loi, F. (2020). Standardized methodology for target surveillance against African Swine Fever. Vaccines, 8(4), 723. 10.3390/vaccines8040723 PMC7761549 33276509

[vms3824-bib-0005] Danzetta, M. L. , Marenzoni, M. L. , Iannetti, S. , Tizzani, P. , Calistri, P. , & Feliziani, F. (2020). African Swine Fever: Lessons to learn from past eradication experiences. A systematic review. Frontiers in Veterinary Science, 7, 296. 10.3389/fvets.2020.00296 32582778 PMC7296109

[vms3824-bib-0006] EFSA AHAW Panel (EFSA Panel on Animal Health and Welfare) . (2014). Scientific opinion on African swine fever. EFSA Journal, 12(4), 3628, 77 pp. 10.2903/j.efsa.2014.3628

[vms3824-bib-0007] Farez, S. , & Morley, R. S. (1997). Potential animal health hazards of pork and pork products. Revue Scientifique et Technique (International Office of Epizootics), 16(1), 65–78. 9329109 10.20506/rst.16.1.992

[vms3824-bib-0008] Guinat, C. , Gubbins, S. , Vergne, T. , Gonzales, J. L. , Dixon, L. , & Pfeiffer, D. U. (2016). Experimental pig‐to‐pig transmission dynamics for African swine fever virus, Georgia 2007/1 strain. Epidemiology and Infection, 144(1), 25–34. 10.1017/S0950268815000862 25989921 PMC4697298

[vms3824-bib-0009] Italian Ministry of Health (2019). Peste Suina Africana – Piano di Sorveglianza e prevenzione in Italia e Piano di Eradicazione in Regione Sardegna per il 2020. http://www.izsum.it/IZSUM/Common/pages02/wfDettListaDoppia.aspx?EDIT=False&ID=24264&IDMAP=512 (Accessed 19 October 2021)

[vms3824-bib-0010] Jori, F. , Chenais, E. , Boinas, F. , Busauskas, P. , Dholllander, S. , Fleischmann, L. , Olsevskis, E. , Rijks, J. M. , Schulz, K. , Thulke, H. H. , Viltrop, A. , & Stahl, K. (2020). Application of the World Cafè method to discuss the efficiency of African swine fever control strategies in European wild boar (*Sus scrofa*) populations. Preventive Veterinary Medicine, 185, 105178. 10.1016/j.prevetmed.2020.105178 33099152

[vms3824-bib-0011] King, D. P. , Reid, S. M. , Hutchings, G. H. , Grierson, S. , Wilkinson, P. J. , Dixon, L. K. , Bastos, D. S. , & Drew, T. W. (2003). Development of a TaqMan PCR assay with internal amplification control for the detection of African swine fever virus. Journal of Virological Methods, 107(1), 53–61. 10.1016/S0166-0934(02)00189-1 12445938

[vms3824-bib-0012] Laddomada, A. , Rolesu, S. , Loi, F. , Cappai, S. , Oggiano, A. , Madrau, M. P. , Sanna, M. S. , Pilo, G. , Bandino, E. , Brundu, D. , Cherchi, S. , Masala, S. , Marongiu, D. , Bitti, G. , Desini, P. , Floris, V. , Mundula, L. , Carboni, G. , Pittau, M. , … Sgarangella, F. (2019). Surveillance and control of African Swine Fever in free‐ranging pigs in Sardinia. Transboundary Emerging Diseases, 66, 1114–1119. 10.1111/tbed.13138 30715791 PMC6849606

[vms3824-bib-0013] Loi, F. , Cappai, S. , Coccollone, A. , & Rolesu, S. (2019). Standardized risk analysis approach aimed to evaluate the last African Swine Fever eradication program performance, in Sardinia. Frontiers in Veterinary Science, 6, 299. 10.3389/fvets.2019.00299 31572734 PMC6753231

[vms3824-bib-0014] Loi, F. , Cappai, S. , Laddomada, A. , Feliziani, F. , Oggiano, A. , Franzoni, G. , Rolesu, S. , & Guberti, V. (2020). Mathematical approach to estimating the main epidemiological parameters of African Swine Fever in wild boar. Vaccines, 8, 521. 10.3390/vaccines8030521 PMC7563513 32932614

[vms3824-bib-0015] McKercher, P. D. , Hess, W. R. , & Hamdy, F. (1978). Residual viruses in pork products. Applied and Environmental Microbiology, 35(1), 142–145. 564162 10.1128/aem.35.1.142-145.1978 PMC242793

[vms3824-bib-0016] Mebus, C. A. , House, C. , Ruiz Gonzalvo, F. , Pineda, J. M. , Tapiador, J. , Pire, J. J. , Bergada, J. , Yedloutschnig, R. J. , Sahu, S. , Becerra, V. , & Sanchez‐Vizcaino, J. M. (1993). Survival of foot‐and‐mouth disease, African swine fever, and hog cholera viruses in Spanish Serrano cured hams and Iberian cured hams, shoulders and loins. Food Microbiology, 10(2), 133–143. 10.1006/fmic.1993.1014

[vms3824-bib-0017] Mebus, C. , Arias, M. , Pineda, J. M. , Tapiador, J. , House, C. , & Sanchez Vizcaino, J. M. (1997). Survival of several porcine viruses in different Spanish drycured meat products. Food Chemistry, 59(4), 555–559. 10.1016/S0308-8146(97)00006-X

[vms3824-bib-0018] Mur, L. , Sanchez‐Vizcaıno, J. M. , Fernandez‐Carrion, E. , Jurado, C. , Rolesu, S. , Feliziani, F. , Laddomada, A. , & Martínez‐López, B. (2017). Understanding African Swine Fever infection dynamics in Sardinia using a spatially explicit transmission model in domestic pig farms. Transboundary and Emerging Diseases, 65, 123–134. 10.1111/tbed.12636 28296281

[vms3824-bib-0019] National Contingency Plan http://www.salute.gov.it/imgs/C_17_pagineAree_1670_listaFile_itemName_0_file.pdf (Accessed 19 October 2021)

[vms3824-bib-0020] OIE (World Organisation for animal health) . (2019). Terrestrial animal health code. https://www.oie.int/en/what‐we‐do/standards/codes‐and‐manuals/terrestrial‐code‐online‐access/ (Accessed 19 October 2021)

[vms3824-bib-0021] Regulation (EU) (2016) 2016/429 of the European Parliament and of the Council of 9 March 2016 on transmissible animal diseases and amending and repealing certain acts in the area of animal health (‘Animal Health Law’). https://eur‐lex.europa.eu/eli/reg/2016/429/2021‐04‐21 (Accessed 19 October 2021)

[vms3824-bib-0022] Sánchez‐Vizcaíno, J. M. , Martínez‐López, B. , Martínez Avilés, M. , Martins, C. , Boinas, F. , Vial, L. , Michaud, V. , Jori, F. , Etter, E. , Albina, E. , & Roger, F. (2009). Scientific review on African Swine Fever. EFSA Supporting Publication, 6(8), EN–5. 1–141. 10.2903/sp.efsa.2009.EN-5

[vms3824-bib-0023] Schulz, K. , Conraths, F. J. , Blome, S. , Staubach, C. , & Sauter‐Louis, C. (2019). African Swine Fever: Fast and furious or slow and steady? Viruses, 11(9), 866. 10.3390/v11090866 PMC6783890 31533266

[vms3824-bib-0024] USDA Statement on Confirmation of African Swine Fever in the Dominican Republic , 28 July (2021a). https://www.aphis.usda.gov/aphis/newsroom/news/sa_by_date/sa‐2021/asf‐confirm (Accessed 22 December 2021)

[vms3824-bib-0025] USDA Statement on Confirmation of African Swine Fever in the Dominican Republic , 21 September (2021b). https://www.aphis.usda.gov/aphis/newsroom/stakeholder‐info/sa_by_date/sa‐2021/sa‐09/asf‐haiti (Accessed 22 December 2021)

[vms3824-bib-0026] VETINFO . https://www.vetinfo.it/ (Accessed 19 October 2021)

[vms3824-bib-0027] WORKING DOCUMENT SANTE/7113/2015 (2015). “Strategic approach to the management of African Swine Fever for the EU” Rev 12 ‐29.04.2020. https://ec.europa.eu/food/system/files/2020‐04/ad_control‐measures_asf_wrk‐doc‐sante‐2015‐7113.pdf (Accessed 19 October 2021)

